# Clinical Profile and Complications of Paracentesis in Refractory Ascites Patients With Cirrhosis

**DOI:** 10.14740/gr661w

**Published:** 2015-07-22

**Authors:** Sreenivasa Rao Sudulagunta, Mahesh Babu Sodalagunta, Shiva Kumar Bangalore Raja, Hadi Khorram, Mona Sepehrar, Zahra Noroozpour

**Affiliations:** a Dr.B.R.Ambedkar Medical College, Bangalore, India; b K S Hegde Medical College, India; c Department of Medicine, Dr.B.R.Ambedkar Medical College, Bangalore, India; d Otolaryngology Department, Dr.B.R.Ambedkar Medical College, Bangalore, India; e Baptist Hospital, Bangalore, India

**Keywords:** Cirrhosis, Ascites, Paracentesis, Pig tail catheter, Ultrasound, Dyslipidemia

## Abstract

**Background:**

Large volume paracentesis is found to be safer and more effective for the treatment of tense ascites compared with larger-than-usual doses of diuretics according to studies. The objectives of the study was to evaluate patients with refractory ascites regarding clinical profile, technique of paracentesis, complications, amount of ascites drained, prognosis and co-morbid conditions associated with it.

**Methods:**

Retrospective study was performed including patients between January 2011 and December 2013 with data pooled from total of five hospitals. A total of 4,389 paracenteses were performed on the 1,218 patients with a mean volume of 4,900 ± 2,795 mL ascitic fluid drained. Blind technique, ultrasound-guided technique of paracentesis and pig tail catheter drainage were evaluated. Diabetes mellitus data from available patients and data regarding co-morbidities were analyzed. Coagulation abnormalities in patients were studied.

**Results:**

Study group age ranged from 34 to 79 years, and alcohol is the main cause of cirrhosis. Dyslipidemia was observed in 1,080 patients (88.66%). At the time of inclusion in the study, 40% of the patients had ≥ 2 other cirrhosis-related complications and 20% of the study population had ≥ 3 complications. Early complications occurred in 27.5% (337) of patients and late complications constituted 16.83% (205 patients).

**Conclusions:**

Even with abnormal coagulation, paracentesis is a safe procedure. But significant co-morbidities should be addressed with care in cirrhosis patients. Ultrasound guidance during the procedure whenever required should be encouraged.

## Introduction

Refractory ascites is defined as ascites that does not recede or that recurs shortly after therapeutic paracentesis, despite sodium restriction and diuretic treatment. Cirrhosis is defined histologically as a diffuse hepatic process characterized by fibrosis and the conversion of normal liver architecture into structurally abnormal nodules [1]. Cirrhosis can present with varied complications at one end of the spectrum that is asymptomatic, whereas on other end, complications such as hepatic encephalopathy, gastroesophageal varices and ascites. One of the danger signs of decompensated liver disease is refractory ascites, having a prevalence of 5-10% in patients with ascites [2] and survival rate as low as 50% at 6 - 12 months post-diagnosis [3-5].

Refractory ascites refers to the inability to mobilize ascitic fluid with diagnostic criteria illustrated by Moore et al [6] (Table 1). Even when patients are on a sodium-restricted diet (≤ 90 mmol/day), excretion of sodium is less than what they consume [3]. Despite giving on maximum doses of diuretics (furosemide 160 mg and spironolactone 400 mg) for at least 1 week, ascites persists with a mean weight loss of less than 0.8 kg over 4 days [3]. Multiple early recurrences of ascites, usually seen within 4 weeks of fluid mobilization, may be the first presentation of refractory ascites in many patients [3].

**Table 1:** Diagnostic Criteria of Refractory Ascites

1) Lack of response to maximal doses of diuretic for at least 1 week
2) Diuretic-induced complications in the absence of other precipitating factors
3) Early recurrence of ascites within 4 weeks of fluid mobilization
4) Persistent ascites despite sodium restriction
5) Mean weight loss < 0.8 kg over 4 days
6) Urinary sodium excretion less than sodium intake

Adapted from Moore et al.

Refractory ascites has a poor prognosis, and complications are illustrated in Table 2. Several large randomized controlled trials have shown that repeated large volume paracentesis (4 - 6 L) is safer procedure. It is considered more effective in tense ascites compared to larger-than-usual doses of diuretics [7-10]. Benefits observed were lower incidence of systemic and hemodynamic disturbance, electrolyte abnormalities, renal impairment and encephalopathy compared with diuretic therapy [7], also with improvement in cardiac output, lung volumes and reductions in intra-abdominal, portal intra-thoracic and pulmonary pressures.

**Table 2:** Clinical Implications of Refractory Ascites

1) Dilutional hyponatremia
2) Hepatorenal syndrome
3) Spontaneous bacterial peritonitis
4) Hepatic hydrothorax
5) Spontaneous bacterial empyema
6) Umbilical hernia

### Aim of the study

The aim of the study was as follows: 1) evaluate the complications arising from large volume paracentesis; 2) impact of the choice of needle type and usage of ultrasound in paracentesis; 3) amount of drained ascitic fluid and its role in complications; 4) prognosis of the patients who underwent the procedure.

## Materials and Methods

Retrospective study was performed including patients between January 2011 and December 2013. These data were pooled from total of five hospitals with consent taken from all of them. Inclusion criteria were cirrhosis and presence of refractory ascites requiring therapeutic paracentesis. Refractory ascites was diagnosed by the Moore et al criteria illustrated in Table 1. Diagnosis of cirrhosis was based on clinical history, examination, ultrasound, CT abdomen, liver histology and laboratory data.

Exclusion criteria included patients with other co-morbid illnesses cancer, pancreatitis, heart failure, protein losing enteropathies and genetic disorders. A total of 875 patients were excluded due to unavailability of sufficient data of all investigations. The 1,218 patients were included in the study, and data were analyzed using information recorded in each procedure i.e., coagulation parameters prior to paracentesis, volume of drained ascitic fluid, ultrasound guidance, needle usage and type, pig tail catheter usage, immediate complications and dose of infused albumin.

The primary endpoint was occurrence of complications classified as: 1) early or immediate complications including ascitic fluid leak from the puncture site, local bleeding, technical problems (e.g., need for repuncture, slipping of the catheter from the abdominal wall, incomplete procedure) or complications requiring hospitalization within 7 days after the procedure; 2) other serious complications included hepatorenal syndrome (HRS), hepatic encephalopathy, spontaneous bacterial peritonitis (SBP) and large hematomas. The diagnosis of confirmed SBP requires an elevated ascitic fluid absolute polymorphonuclear leukocyte (PMN) count of at least 250 cells/mm^3^ (0.25 × 10^9^/L) and a positive ascitic fluid bacterial culture without an obvious intra-abdominal source of infection. SBP is considered if it occurred within 30 days of procedure.

Secondary endpoints were defined as: 1) the volume of ascitic fluid drained expressed as mean volume in mL; and 2) patient’s prognosis, which was defined as the patient’s status on December 1, 2014. The cause of death was determined from the patient’s record. P-values below 0.05 were considered significant, and 95% confidence interval (CI) was used. Statistical analysis was done with *t*-test, Chi-square test and Fischer’s exact test.

## Results

Study group age ranged from 34 to 79 years and alcohol is the main cause of cirrhosis. Males are predominantly affected. Characteristics of patients were illustrated in Table 3. Males constitute 97% of the study group and females about 3%. The 87% of the study population is above the age of 50 years. In patients with diabetes mellitus 300 patients’ data were available. Poor control of diabetes was observed with about 70% of patients with glycosylated hemoglobin (HBA1C) more than 7%, and HBA1C mean was 8.3 ± 2.09. The 100 patients (33.33%) out of 300 patients with diabetes had diabetes for more than 10 years.

**Table 3:** Patient Characteristics

Category	Number (%)
Age, median (range)	56 (34 - 79)
Age category (years)	
< 50	204 (17%)
50 - 59	602 (50.16%)
60 - 69	214 (17.83%)
70 - 79	198 (16.25%)
> 80	0
Gender	
Male	1,183 (97.126%)
Female	35 (2.873%)
HbA1c, mean (available for 300 patients)	8.3 ± 2.09
HbA1c category	
< 6%	29 (9.66%)
6-6.9%	60 (20%)
7-7.9%	120 (40%)
8-8.9%	69 (23%)
> 9%	22 (7.33%)
Diabetes duration > 10 years	100 (33.33%)
Dyslipidemia	1,080 (88.66%)
Abnormal LDL	1,019 (83.66%)
Abnormal HDL	721 (59.19%)
High total cholesterol	1,001 (82.18%)
Triglycerides	903 (74.13%)
Patients taking statins	404 (33.16%)

Dyslipidemia was observed in 1,080 patients (88.66%) which is statistically significant than normal population (P < 0.001). In patients with dyslipidemia, abnormal low density lipoprotein (LDL) was found in 1,019 patients (83%), abnormal high density lipoprotein (HDL) was found in 721 patients (59%) and hypertriglyceridemia in 903 patients (74%). Patients taking statins constituted only 33% of patients (404), which is statistically significant (P < 0.001) and alarming.

Alcoholism constituted 94.5% (1,151 patients) of the cause of cirrhosis of liver and ascites. Hepatitis B was associated with 42 patients (3.44%), hepatitis C with 16 patients (1.31%) and cryptogenic cirrhosis in 0.73% (nine) of patients. Majority of the patients included in the study had cirrhosis in advanced stage with a high Child-Pugh score median score of 10 (range 8 - 15) and mean 10.5, 95% CI. The 73% belong to Child-Pugh class C. Clinical and laboratory data are illustrated in Table 4.

**Table 4:** Clinical and Laboratory Data

Age, years, median (range)	56 (34 - 79)
Sex	n (%)
Male	1,183 (97.126%)
Female	35 (2.873%)
Etiology	n (%)
Alcoholic	1,151 (94.49%) (F: 28; M: 1,123)
Hepatitis C-related	16 (1.313%) (F: 2; M: 14)
Hepatitis B-related	42 (3.448%) (F: 3; M: 39)
Cryptogenic	9 (0.738%) (F: 2; M: 7)
Serum albumin, g/dL	26 (14.3 - 38.0)
Serum bilirubin, μmol/L, median (range)	31 (3.0 - 304.0)
Serum creatinine, μmol/L, median (range)	87 (37 - 379)
INR, median (range)	1.5 (1.0 - 3.0)
Child-Pugh score, median (range)	10 (8 - 15)
Model for end-stage liver disease (MELD) score, median (range)	14.5 (6.2 - 28.9)

At the time of inclusion in the study, 40% of the patients had ≥ 2 other cirrhosis-related complications, and 20% of the study population had ≥ 3 complications including esophageal varices, hepatic encephalopathy, hepatocellular carcinoma, hepatorenal syndrome, hepatopulmonary syndrome and spontaneous bacterial peritonitis. Co-morbid conditions were associated with > 50% of patients in study group (Table 5). The 16.5% patients (209) had ischemic heart disease (IHD), 12.5% (153) had chronic kidney disease (CKD), 8.5% (104) patients had cerebrovascular disease and 8% (99) had chronic obstructive pulmonary disease. Patients with ≥ 2 co-morbidities constituted 23.5% (287 patients) of the study group.

**Table 5:** Co-morbid Conditions in Patients

Co-morbidities	Number (%)
CKD	153 (12.56%)
IHD	209 (16.50%)
Cerebrovascular disease	104 (8.53%)
COPD	99 (8.12%)
≥ 2 co-morbidities	287 (23.56%)

A total of 4,389 paracenteses were performed on the 1,218 patients with a mean volume of 4,900 ± 2,795 mL ascitic fluid drained. Early complications occurred in 27.5% (337) of patients which are illustrated in Table 6. Early complications observed were incomplete drainage, pain at puncture site, local bleeding, hypotension, increased shortness of breath (SOB), re-punctures done, slipping of catheter used for tapping and leakage of ascitic fluid from puncture site. Late complications constituted 16.83% (205 patients). Late complications observed were fever, abdominal hematoma, hepatic encephalopathy, spontaneous bacterial peritonitis, hepatorenal syndrome and hepatopulmonary syndrome (Table 6). Out of these complications 7% were due to technical errors during performance or after the procedure.

**Table 6:** Variables and Paracenteses Percentages

Calculated variables	Paracenteses (n = 4,389)
Early complications	n (%)
Incomplete drainage	69 (5.66%)
Pain at puncture site	57 (4.67%)
Local bleeding	35 (2.87%)
Hypotension	51 (4.18%)
Increased SOB	30 (2.46%)
Re-punctures done	32 (2.62%)
Slipping of catheter used for tapping	24 (1.97%)
Leakage of ascitic fluid from puncture site	39 (3.20%)
Total	337 (27.66%)
Late complications	n (%)
Fever	23 (1.88%)
Abdominal hematoma	30 (2.46%)
Hepatic encephalopathy	88 (7.22%)
Spontaneous bacterial peritonitis	32 (2.62%)
Hepatorenal syndrome	17 (1.39%)
Hepatopulmonary syndrome	15 (1.23%)
Mean volume ± SD of drained ascitic fluid, mL	4,900 ± 2,795 mL
Total	205 (16.83%)

Intravenous catheter was used in 4,189 paracenteses with 4,089 (93.16%) being blind procedures where the dependent portion of ascites was drained (Table 7) [11]. The 100 (2.27%) paracenteses were performed using ultrasound guidance. The complication rate in ultrasound-guided procedure was 0% compared to blind procedure which is statistically significant (P < 0.001). In all these ultrasound-guided procedures paracentesis needle was used. In 200 (4.55%) procedures pig tail catheter was used with higher rate of complications compared to intravenous catheter usage procedures, although it was not statistically significant. Early complications with pig tail catheter were 37 (18.5%) compared to intravenous catheter procedure with 300 (7.33%). Late complications with pig tail catheter were 8% compared to 5% of intravenous catheter procedure.

**Table 7:** Ascitic Tap Procedure Followed in the Study [11]

Explain the procedure to the patient, including risks, and obtain consent
Position the patient, usually in the supine position with the head of the bed elevated to allow fluid to accumulate in the patient’s lower abdomen
Position of the tap
Locate area of flank dullness lateral to the rectus abdominis muscle and go approximately 5 cm superior and medial to the anterior superior iliac spines
Avoid the inferior epigastric vessels which run up the side of the rectus abdominis to anastomose with the superior epigastric vessels coming down
Avoid the pelvic area, solid tumor masses, prominent superficial veins (caput medusa) and scars (may have collateral vessels close by or adherent bowel beneath)

## Discussion

Paracentesis is a word derived from Greek κεντάω (“to pierce”), a common procedure done in outpatient department and medical wards. According to recent studies mild hematological abnormalities do not increase risk of bleeding significantly [12, 13]. Risk of bleeding increases with the following [14]: 1) prothrombin time > 21 s; 2) international normalized ratio (INR) > 1.6 and 3) platelet count < 50,000/mm^3^. Acute abdomen requiring surgery is an absolute contraindication. Relative contraindications are pregnancy, distended urinary bladder, abdominal wall cellulitis, distended bowel and intra-abdominal adhesions [15].

Intravenous albumin infusions have been shown in recent studies to reduce the rate of complications by preventing post-paracentesis-induced circulatory dysfunction (PICD). In spite of the benefits, still procedure is associated with many complications [16]. Complications found in this study were similar to other retrospective and prospective studies, even though few complications were minimal such as bleeding, slippage of catheter were less common [17, 18].

Late and serious complications associated with paracentesis were hepatorenal syndrome, hepatopulmonary syndrome, spontaneous bacterial peritonitis and hepatic encephalopathy. These complications were observed but uncommonly in the study.

Higher incidence of complications with pig tail catheter compared to intravenous catheter drainage of ascitic fluid was observed. This observation is reinforced by other studies in 2001 by Schlottmann et al and Shaheen and Grimm, which found a significantly lower rate of re-punctures (1:6, P = 0.046) when using a special paracentesis needle [19, 20]. But the mean ascitic fluid was 250 mL more drained through pig tail catheter compared to intravenous catheter needle. The high incidence of complications in the pig tail catheter group may be due to excess and rapid drainage.

We found that ultrasound-guided ascitic tap is safer than blind procedure and using pig tail catheter. Using paracentesis needle is also accounted for the benefit in the patients who have undergone ultrasound-guided ascitic fluid drainage. Ultrasound-guided paracentesis is particularly useful with minimal-to-moderate ascites, access is difficult and complications were expected due to drainage of the ascitic fluid described by Gottardi et al [21]. Few cases are reported regarding deaths due to intra-peritoneal hemorrhage due to paracentesis even under ultrasound guidance.

Our study found these significant observations. 1) Paracentesis is a relatively safe procedure with low complication rate. 2) Ultrasound-guided paracentesis was observed to have low complication rate even though blind paracentesis procedure is relatively safe with less complications. 3) No deaths were observed during and up to 1 month of paracentesis procedure attributable to the procedure. 4) Even though around 50% of patients had aberrant coagulation parameters, the complications of bleeding were significantly low. 5) As it is well known, alcohol is the major cause of cirrhosis of liver. 6) Poor control of diabetes was observed among the cirrhotic patients undergoing paracentesis. 7) Co-morbidities like cerebrovascular disease, IHD, and CKD are significantly higher in cirrhotic patients. 8) Almost more than 90% patients were dyslipidemic with low statins usage which should be addressed as the mortality increases.

### Conclusions

Even with abnormal coagulation, paracentesis is a safe procedure. But significant co-morbidities should be addressed with care in cirrhosis patients. Ultrasound guidance during the procedure whenever required should be encouraged.
